# Diet and behavioral habits related to oral health in eating disorder patients: a matched case-control study

**DOI:** 10.1186/s40337-020-0281-z

**Published:** 2020-02-27

**Authors:** Ann-Katrin Johansson, Claes Norring, Lennart Unell, Anders Johansson

**Affiliations:** 1 Department of Clinical Dentistry-Cariology, Faculty of Medicine, University of Bergen, Årstadveien 19, 5009 Bergen, Norway; 2 Department of Clinical Neuroscience, Karolinska Institutet, Stockholm, Sweden; 3 School of Health and Medical Sciences, Örebro University and Region Örebro County Council, Örebro, Sweden; 4 Department of Clinical Dentistry–Prosthodontics, Faculty of Medicine, University of Bergen, Bergen, Norway

**Keywords:** Awareness, Behaviors, Diet, Oral hygiene, Risk factors, Soft drinks

## Abstract

**Background:**

Patients suffering from eating disorders (ED) have a substantially increased risk for developing poor oral health. In this regard, dietary habits in combination with obsessive behavior as well as the expression and intensity of the disease are of utmost importance. This study aimed to investigate diet and behavioral habits in patients with ED compared to healthy controls.

**Methods:**

All patients who initiated treatment in an ED clinic during 1 year were invited to participate in the study. Sixty-five patients were admitted out of which 54 agreed to participate: 50 women and 4 men, mean age 21.5 years, range 10–50 years. From a public dental health clinic, 54 sex-and age-matched controls where selected. In all participants a comprehensive questionnaire was completed. ED patients were analyzed with respect to their self-perceived disease state: when they felt “relatively good” (ED-good) and “bad” (ED-bad) as well as if they reported vomiting or not.

**Results:**

The ED-good patients reported significantly higher intake of caffeine-containing and cola light soft drinks and both study groups reported a lower intake of regularly sweetened carbonated drinks compared to controls. ED-bad reported significantly lower intake of number of meal and sweet intake while both study groups brushed their teeth more frequently than controls. As regards awareness of detrimental dietary intake and the possible risk for oral health complications did not differ between patients and controls except that the ED groups were more aware that vomiting and brushing thereafter could damage their teeth. ED patients went less often to the dentist for regular checkups than controls. Vomiting ED patients differed in several of the parameters related to dietary and other behaviors compared to no vomiting subjects. According to regression analyses and compared to healthy controls, predictive variables for ED-good were: higher intake of caffeine containing drinks (OR 1.34, CI 1.10–1.64) and lower intake of regular soft drinks (OR 0.57, CI 0.35–0.94). For ED-bad, lower frequency intake of lunch meals (OR 0.59, CI 0.39–0.88) and sweet biscuits were predictive (OR 0.15, CI 0.05–0.48).

**Conclusions:**

ED patients present a number of dietary and other types of behavior that are potentially harmful for oral health. It is important to retrieve reports on the ED behaviors in both relatively good and bad disease state in order for the medical team to prescribe adequate advice and treatment.

## Plain English summary

Patients suffering from eating disorders have an increased risk for developing poor oral health. In this regard, the fluctuating severity of the disease may be connected to changes in behavior pattern such as more unhealthy way of eating and drinking as well as in harmful oral hygiene habits. This study examined diet and behavioral habits in patients with eating disorders when they felt relatively good or bad in their disease compared to healthy controls.

Depending on the self-perceived disease condition (relatively good or bad), eating disorders patients consume more caffeine-containing and cola light soft drinks, lesser sweet carbonated drinks and number of meals. They also brushed their teeth more frequently but went less often for dental check-up than controls. Predictive factors for being an eating disorder patents were higher intake of caffeine containing drinks, lower intake of sweet soft drinks and biscuits and reduced number of lunch meals. Eating disorder patients present a number of dietary and other types of behavior that are potentially harmful for oral health. It is important to retrieve reports on behaviors in both relatively good and bad disease state in order for the medical team to prescribe adequate advice and treatment.

## Introduction

Patients suffering from eating disorders (ED) such as Anorexia Nervosa (AN), Bulimia Nervosa (BN) and eating disorders otherwise not specified (EDNOS) have a substantially increased risk for developing poor oral health. In this regard, the combination of harmful dietary habits, self-induced vomiting, impaired salivary conditions and less favorable oral hygiene habits will increase the risk for oral diseases such as dental erosion and dental caries( [1]) as well as for temporomandibular disorder (TMD) [2]. In a meta-analysis comprising ten studies, the odds for presenting hyposalivation and dental erosion was about 2–7 times higher in ED patients compared to controls. Dental caries, based on decayed-missing-filled surfaces (DMFS), was in average 3.07 lower in controls compared to those diagnosed with ED [3]. ED are difficult to treat and although many individuals recover in the long term, a prolonged course with recurring relapses and an elevated risk of premature death is not unusual [4, 5].

Dietary habits in combination with obsessive behavior as well as the expression and intensity of the disease are of utmost importance for oral health in ED patients [6]. This connection is especially apparent between bulimic behaviors and dental erosion. The acidic challenge for the teeth in bulimic patients is depending not only on the type of the diet or drinks ingested but also in purging behavior caused by the gastric hydrochloric acid reaching the oral cavity [7–9]. Consumption of regularly sweetened soft drinks and juices will increase the risk for both dental erosion and caries, and artificially sweetened soft drinks, without regular sugar, will increase the risk for dental erosion [10]. In this regard, it has been found that patients with ED use soft drinks with artificial sweeteners more often than controls [11] and it has been suggested that they choose to drink diet drinks in order to control both their appetite and weight. It has even been suggested that monitoring intake of low-calorie diet in ED patients, such as light soft drinks, may be of importance when predicting the treatment outcome of an ED [12, 13]. Besides the choice of dietary products, the pattern of consumption, oral hygiene habits and awareness about possible negative factors for oral health as well as utilization of dental care services are other behaviors that may be of importance. The effect of these behaviors may also be influenced by the common variation in ED symptomatology, with the patient having alternate periods of bad or relatively healthy/good disease state.

The aim of this study is to investigate the above-mentioned behaviors in ED patients during periods when their self-perceived ED status was “relatively good” vs. “bad”, compared to sex- and age-matched healthy controls. The hypothesis of this study is that diet and other behavioral habits differs among ED patients depending on their disease state.

## Materials and methods

### Participant selection

Sixty-five consecutive patients attending the Eating Disorder Clinic, Örebro County Council, Örebro, Sweden during one-year period were invited to participate in the study. Fifty-four of those accepted and a sex and age matched control group was selected from a Public Dental Health Clinic, Örebro, Sweden. All controls were tested for possible ED diagnosis using the Symptom Index of the Eating Disorder Inventory-2 (EDI-2) [14]. Two controls had a risk for ED diagnosis and were offered a referral to a specialized ED clinic. Two new controls were selected and both of them scored negatively for ED by the EDI-2. Detailed information of the selection of participants has been reported elsewhere [1].

### Questionnaire

A questionnaire was created together with the medical personnel working at the ED clinic (Additional file 1). It was tested on a group of five subjects and thereafter reassessed and changed according to the comments from the test persons. The questionnaire finally comprised 196 questions on sociodemographic factors, general and oral health including dietary and oral hygiene habits as well as aspects related to utilization of dental care. It was given to both the ED group and controls except for some questions that were specifically designed for the ED-patients and therefore not relevant for the controls. The ED group was asked to respond to the questions based on their self-perceived ED status, i.e. when they judged their ED condition as “relatively good” (ED-good) or as “bad” (ED-bad). Consequently, all ED patients (*n* = 54) answered the two questions. The same question was always given twice, where the patients had to assess the severity of their ED when they were feeling “relatively good” and “bad”. Example of questions: When you feel (relatively good/bad) in your eating disorder, what do you drink/eat (certain items)? When you feel (relatively good/bad) in your eating disorder, how often do you eat breakfast, lunch, dinner and snacks in between meals? In a similar way, dietary questions were given that included type and amount of drink intake, fruit consumption and intake of sweets, biscuits, hard cheese, milk, yoghurt/sour milk and number of meals. Questions about the participants’ awareness of the possible danger for oral health problems from consuming certain food and drink items were also given for the “relatively good” and “bad” ED states. So were questions about oral hygiene habits, e.g. brushing frequency, proximal cleaning, and usage of tooth paste. Utilization of dental care comprised questions on regular recall frequency, emergency visits, confidence in dental care system and dental fear. Detailed methodology in recordings of oral health status, TMD problems and salivary factors have previously been reported on [1, 2, 15].

### Statistical methods

Statistical Package for the Social Sciences (SPSS version 24.0, IBM SPSS Corp., Armonk, NY, USA). Differences between ED patients (in relatively good and bad state) and controls were calculated with Friedmans test and differences within the three groups with the Wilcoxon Signed Ranks Test as Post Hoc test. Bonferroni correction was applied and a *p*-value of ≤0.017 was therefore considered significant in case of three pairwise comparisons. In addition to comparing the broad ED group with its matched control, McNemar’s test was used for dichotomous data and Wilcoxon signed Rank test for numerical and ordered ordinal data. In addition, the ED patients were divided on those who reported vomiting and those who did not and their responses to the questionnaire were analyzed in the same manner as for ED-good/bad described above.

Variables related to dietary habits between ED (in relatively good and bad state) and controls were analyzed by conditional logistic regression using the nomreg and the Cox procedures. For each of the regression analyses, six independent variables were selected among those found significantly different in bivariate comparisons without consideration to the Bonferroni correction.

## Results

The mean age for both patients and controls was 21.5 years (SD = 6.8, range 10–50; 100 females, 8 males). Regarding ED diagnoses 28% of the patients presented with anorexia nervosa (AN, 14/54), 14% with bulimia nervosa (BN, 8/54) and 58% with eating disorder not otherwise specified (EDNOS, 32/54). The diagnoses were given according to DSM-IV [16]. The mean age at the onset and duration of ED in the study group was 16 years (range 9–26) and 4.5 years (range 0.3–35), respectively.

Vomiting was reported by 25 ED patients and no vomiting by 29 patients. The distribution of ED diagnoses in the vomiting group was, 17 EDNOS, 7 BN and 1 AN. The corresponding figures for the no vomiting group was 15 EDNOS, 13 AN, 1 BN.

### Diet

The questionnaire gave report on different types of drinks and foods consumed in the ED group during self-assessed severity of the disease, i.e. relatively good and bad disease state, both of which was compared to that of responses from healthy controls.

#### Drink consumption

The total intake of soft drinks during relatively good and bad conditions of ED compared to controls is presented in Table 1. Friedman test for multiple comparisons between the three groups showed significant differences in reported intake of cola, other regular (sweetened) carbonated soft drinks, nutrition drinks and milk. Pairwise comparisons between ED-good and matched control applying Bonferroni correction showed that yearly consumption of Cola light was significantly higher in the ED-good group compared to controls (41.4 L vs. 6.6 L; *p* = 0.014) and the same applied about nutrition drinks (25.3 L vs. 0 L; *p* = 0.003). The controls had a significantly higher yearly intake than both ED-good and ED-bad as regard Cola regular (22.6 L vs. 8.7 L vs. 12.3 L; *p* = 0.001 and *p* = 0.005) and other carbonated regular soft drinks (20.7 L vs. 5.3 L vs. 8.0 L; *p* = 0.003 and *p* = 0.010). Milk consumption was significantly higher in controls (107 L/year) compared to ED-bad (69.5 L/year) (*p* = 0.004).

**Table 1:** Total Drink Intake (L/yr) in Relatively Good and Bad Disease State Compared to Healthy Controls

	Relatively good (*n* = 54)		Bad (*n* = 54)		Control (*n* = 54)					
	Mean ± SD	Range	Mean ± SD	Range	Mean ± SD	Range	P	P_1_	P_2_	P_3_
Cola light	41.4 ± 108.0	0–566	63.1 ± 198.7	0–1205	6.6 ± 16.6	0–85	0.019	0.014	NS	NS
Cola regular	8.7 ± 42.4	0–301	12.3 ± 48.8	0–301	22.6 ± 34.4	0–151	0.0001	0.001	0.005	NS
Other carbonated light soft drinks	13.6 ± 75.2	0–547	19.9 ± 89.3	0–548	11.7 ± 37.1	0–183	NS	NS	NS	NS
Other carbonated regular soft drinks	5.3 ± 26.8	0–183	8.0 ± 35.3	0–183	20.7 ± 54.6	0–365	0.0001	0.003	0.01	NS
Sport drinks	0.3 ± 2.3	0–17	0	0	2.4 ± 14.3	0–104	NS	NS	NS	NS
Apple vinegar	0.4 ± 2.0	0–15	0.9 ± 4.3	0–27	0.6 ± 4.7	0–34	NS	NS	NS	NS
Juice	28.8 ± 50.0	0–230	33.0 ± 57.7	0–230	44.7 ± 51.9	0–183	0.03	NS	NS	NS
Nutrition drinks	25.3 ± 55.7	0–219	15.5 ± 50.5	0–274	0	0	0.0001	0.003	0.03	NS
Tea without sugar	86.5 ± 122.4	0–438	70.4 ± 156.5	0–949	40.3 ± 66.8	0–237	NS	0.04	NS	NS
Tea with sugar	16.5 ± 48.8	0–256	17.5 ± 55.3	0–329	20.4 ± 48.3	0–219	NS	NS	NS	NS
Coffee without sugar	96.4 ± 219.3	0–1186	88.3 ± 224.9	0–1186	31.4 ± 81.4	0–329	NS	NS	NS	NS
Coffee with sugar	3.6 ± 22.5	0–164	3.4 ± 22.4	0–164	4.5 ± 25.4	0–183	NS	NS	NS	NS
Milk	89.7 ± 94.3	0–365	69.5 ± 101.6	0–548	107.1 ± 107.9	0–438	0.001	NS	0.004	0.02
Water	342.4 ± 307.0	0–1095	386.1 ± 463.7	0–2008	310.1 ± 254.8	0–1095	NS	NS	NS	NS
Total drink intake (per above)	758.2 ± 452.7	129–2444	794.8 ± 719.9	0–3833	623.9 ± 286.7	0–1462	NS	NS	NS	NS

P refers to differences between the three groups by Friedman test. P_1_ refers to difference between relatively good vs. control; P_2_ bad vs. control; P_3_ relatively good vs. bad (Wilcoxon Signed Rank Test)

Descriptive data and inferential analyzes for the vomiting and no vomiting groups are described in the Additional file 2: Tables S1 and S2. Vomiting ED patients reported significantly higher intake of Cola light in ED-good (79.9 L) compared to matched controls (6.8 L) (*p* = 0.003). No differences as regard Cola light consumption were found in the no vomiting group. In the no vomiting group, Cola regular consumption was significantly higher in the controls (27.3 L) compared to both ED-good (11.0 L) and bad (10.8 L) (*p* = 0.004 and *p* = 0.005, respectively. No differences in Cola regular intake were found the vomiting group. No differences in nutrition drink intake were found in the vomiting group, while ED-good presented significantly higher intake (32 L) compared to controls (0 = L) (*p* = 0.01) in the no vomiting group.

Grouping of the different types of drinks is shown in Table 2 and Friedman comparisons showed significant differences in intake of total soft drinks, carbonated soft drinks, regular soft drinks and caffeine-containing drinks (cola-type drink, tea and coffee). In pairwise comparisons using Bonferroni correction the controls reported significantly higher intake of carbonated regular soft drinks than both ED-good and bad groups (43.3 vs. 14.0 vs. 20.4; *p* = 0.0001 and *p* < 0.001). Yearly consumption of caffeine-containing drinks was significantly higher in ED-good than in controls (251.4 L vs. 125.8 L; *p* = 0.001).

**Table 2:** Soft Drink Consumption (L/yr) in Relatively Good and Bad Disease State Compared to Healthy Controls

	Relatively good (n = 54)		Bad (n = 54)		Control (*n* = 54)					
	Mean ± SD	Range	Mean ± SD	Range	Mean ± SD	Range	P	P_1_	P_2_	P_3_
Total soft drinks^1^	97.5 ± 175.4	0–1029	135.8 ± 292.8	0–1752	108.8 ± 97.0	0–511	0.004	NS	NS	NS
Total carbonated soft drinks	68.9 ± 166.7	0–1029	103.4 ± 277.7	0–1752	61.6 ± 69.8	0–365	0.03	NS	NS	NS
Total carbonated light soft drinks	54.9 ± 162.9	0–1029	83.0 ± 274.3	0–1752	18.3 ± 43.4	0–182	NS	NS	NS	NS
Total carbonated regular soft drinks	14.0 ± 52.8	0–313	20.4 ± 70.2	0–365	43.3 ± 59.8	0–365	0.0001	0.0001	0.001	NS
Total caffeine-containing drinks^2^	251.4 ± 254.6	0–1186	253.7 ± 367.3	0–1642	125.8 ± 123.2	0–523.0	0.047	0.001	0.047	NS

P refers to differences between the three groups by Friedman test. P_1_ refers to difference between relatively good vs. control; P_2_ bad vs. control; P_3_ relatively good vs. bad (Wilcoxon Signed Rank Test)

^1^Carbonated beverages (light and regular), sport drinks, juice

^2^Cola-type drinks, tea, coffee

In the no vomiting group, total soft drink intake was significantly higher in the controls (107 L) compared to ED-good (59.0 L) (*p* = 0.01). The same applied to carbonated regular soft drink intake in the no vomiting group where controls reported significantly higher intake (52.6 L) compared to ED-good (18.2 L) and bad (17.6 L) (*p* = 0.002). In the vomiting group, controls reported significantly higher intake of total carbonated regular soft drinks (32.5 L) compared to ED-good (9.1 L) (*p* = 0.01). Intake of caffeine-containing drinks was higher in ED good compared to controls in the vomiting group (330 L vs. 144 L) (*p* = 0.009). No differences were found as regards caffeine drinks in the no vomiting subjects (see Additional file 2).

#### Food habits

Number of meals was significantly different between the three groups at all types of reported occasions (Table 3). In pairwise tests, the ED-good group did not present any significant difference to the controls in any of the types of meal while the ED-bad group did so: weekly numbers of breakfast, lunch and dinner meals were all significant lower in the ED-bad group compared to controls (*p* = 0.002 to *p* = 0.0001). The same applied in comparisons between the two ED-groups.

**Table 3:** Eating Habits in Relatively Good and Bad Disease State Compared to Healthy Controls

	Relatively good (*n* = 54)		Bad (n = 54)		Control (*n* = 54)					
	Mean ± SD	Range	Mean ± SD	Range	Mean ± SD	Range	P	P_1_	P_2_	P_3_
Number of meals/day	3.8 ± 1.5	1–7	3.0 ± 1.8	0–7	3.4 ± 1.0	1–5.5	0.002	NS	NS	0.001
Number breakfast/week	6.0 ± 2.1	0–7	4.8 ± 2.9	0–7	6.3 ± 1.5	1–7	0.0001	NS	0.0001	0.001
Number of lunch/week	6.0 ± 1.9	0–7	4.6 ± 2.6	0–7	6.5 ± 1.3	1–7	0.0001	NS	0.0001	0.0001
Number of dinner/week	6.5 ± 1.2	2–7	5.0 ± 2.6	0–7	6.4 ± 1.5	1–7	0.0001	NS	0.002	0.0001
Number of in-between meals/week	6.3 ± 3.5	0–17	4.9 ± 6.1	0–40	5.1 ± 3.0	0–14	0.02	NS	NS	0.02

P refers to differences between the three groups by Friedman test. P_1_ refers to difference between relatively good vs. control; P_2_ bad vs. control; P_3_ relatively good vs. bad (Wilcoxon Signed Rank Test)

In the vomiting group and compared to controls, ED-bad had significantly lesser frequent intake of breakfast (3.9 vs. 6.0 times), lunch (3.7 vs. 6.6 times) and dinner (4.4 vs. 6.4) (*p* = 0.008, *p* = 0.001 and p = 0.001, respectively). Comparisons between ED-good and bad showed that the latter had had significantly lesser intake than ED-good at all meal occasions except for in-between meals (*p* = 0.016 to *p* = 0.005). Both ED-good and bad in the no vomiting presented no differences compared to controls in number of meals/breakfast/lunch/dinner/in between meals. Comparison between ED-good and bad revealed that the latter had significantly lesser frequent intake of total meals/day (3.3 vs. 3.8 times) and lunch/day (5.4 vs. 6.6 times) (*p* = 0.002 and p = 0.005, respectively) (see Additional file 2).

Regarding fruit consumption, the only significant difference detected was consumption of weekly intake of apples where the ED-good consumed more than controls (6.6 vs. 3.2; *p* = 0.006) (Table 4). In the vomiting group and compared to the matched controls, no differences were found while in the no vomiting group ED-bad consumed significantly more (6.9 apples/week) compared to controls (2.6 apples/week) (*p* = 0.01).

**Table 4:** Fruit Intake (Number/Week) in Relatively Good and Bad Disease State Compared to Healthy Controls

	Relatively good (*n* = 54)		Bad (n = 54)		Control (n = 54)					
No. of fruit intake/week	Mean ± SD	Range	Mean ± SD	Range	Mean ± SD	Range	P	P_1_	P_2_	P_3_
Apples	6.6 ± 8.6	0–39	6.6 ± 13.0	0–70	3.2 ± 4.7	0–28	0.07*	0.006	NS	NS
Pears	2.3 ± 4.2	0–18	2.9 ± 8.0	0–49	0.9 ± 1.7	0–7	NS	NS	NS	NS
Citrus fruits	4.4 ± 6.6	0–28	3.8 ± 8.8	0–53	2.1 ± 4.3	0–21	NS	NS	NS	NS
Bananas	4.4 ± 9.4	0–64	3.1 ± 5.7	0–28	3.6 ± 4.4	0–25	0.04	NS	NS	NS

P refers to differences between the three groups by Friedman test. P_1_ refers to difference between relatively good vs. control; P_2_ bad vs. control; P_3_ relatively good vs. bad (Wilcoxon Signed Rank Test)

*Tendency for significance

Sweets, sweet biscuits/buns and hard cheese were all significantly different in multiple comparisons between the three groups (*p* = 0.002 to *p* = 0.0001) while the consumption of yoghurt/sour milk was not. In pairwise tests, both ED-good and bad groups reported significantly more often “never or seldom intake” of sweet biscuits/buns than the controls (46.3% vs. 66.7% vs. 18.5%; *p* = 0.004 and p = 0.0001). The ED-bad group had lesser frequent intake of sweets and 51.9% reported intake “never or seldom” compared to controls of 16.7% (*p* = 0.001) and the same applied for hard cheese (58.5% vs. 32.1%, *p* = 0.003) (Table 5).

**Table 5:** Percentage Distribution of Intake of Dietary Items

	Relatively good (n = 54)		Bad (n = 54)		Control (*n* = 54)						
	1	2	1	2	1	2	P	P_1_	P_2_	P_3_	
Sweets	37.0	63.0	51.9	48.1	16.7	83.3	0.0001	0.03	0.001	NS	
Sweet biscuits, buns	46.3	53.7	66.7	33.3	18.5	81.5	0.0001	0.004	0.0001	0.04	
Hard cheese	51.9	48.1	58.5	41.5	32.1	67.9	0.002	NS	0.003	NS	
Yoghurt/sour milk	20.4	79.6	35.2	64.8	20.4	79.6	NS	NS	NS	NS	

1 = Never or seldom; 2 = One to several intake/month, week or day. P refers to differences between the three groups by Friedman test. P_1_ refers to difference between self-perceived relatively good vs. control; P_2_ bad vs. control; P_3_ relatively good vs. bad (McNemar’s Test)

In the vomiting group, no significant differences were found as regards intake of sweets, sweet biscuits/buns and yoghurt/sour milk but hard cheese was consumed more seldom in in the ED-bad group (*p* = 0.01). The controls in the no vomiting group consumed significantly more sweets/sweet biscuits, buns than the ED-bad (*p* = 0.002 and p = 0.001, respectively) (see Additional file 2).

#### Oral hygiene habits

Number of daily tooth brushing differed significantly between the three groups (*p* = 0.016). In pairwise tests, none of the differences reached statistical significance (*p* > 0.017) and neither did length of tooth brushing or amount of tooth paste used (Table 6). Multiple comparisons of time related brushing showed statistical significances in morning, evening and after meal brushing (*p* = 0.039 to *p* = 0.004) but in the pairwise tests only evening brushing turned out be statistically significant in that ED-bad reported less frequent brushing than controls (85% vs. 100%, *p* = 0.008) (data not shown). Responses relating proximal cleaning, rinsing and type of solution after brushing and use of saliva stimulating agents did not differ between ED groups and controls (data not shown).

**Table 6:** Oral Hygiene Habits in Relatively Good And Bad Disease State Compared to Healthy Controls

	Relatively good (n = 54)		Bad (n = 54)		Control (n = 54)					
	Mean ± SD	Range	Mean ± SD	Range	Mean ± SD	Range	P	P_1_	P_2_	P_3_
No. of brushing times/day	2.3 ± 0.7	1–5.5	2.6 ± 1.5	0–10	2.1 ± 0.5	1–3.5	0.016	0.026	0.018	NS
Toothbrushing min/time	3.3 ± 3.3	1–22.5	3.5 ± 3.7	0.5–22.5	2.9 ± 1.6	1–7.5	NS	NS	NS	NS
Cm tooth paste /brushing	1.5 ± 0.9	0.3–6.5	1.5 ± 0.92	0.3–6.5	1.6 ± 0.95	0.2–5	NS	NS	NS	NS

P refers to differences between the three groups by Friedman test. P_1_ refers to difference between relatively good vs. control; P_2_ bad vs. control; P_3_ relatively good vs. bad (Wilcoxon Signed Rank Test)

In comparisons between the vomiting and no vomiting group, the only significant finding was that ED-good patients in the no vomiting group brushed their teeth more frequently than the controls (2.4 vs. 2.0 times/day) (*p* = 0.015) (see Additional file 2).

#### Awareness

No differences between the groups regarding the perceived danger to the teeth of different food item (sour drinks and fruits), brushing after a dietary acidic challenge to the oral environment were found (data not shown). However, ED patients were significantly more aware of that vomiting may damage their teeth (100% vs. 87%, *p* = 0.008) and that tooth brushing after vomiting may create dental damage (71% vs. 35%, *p* = 0.001).

#### Utilization of dental care

ED patients reported that they visit the dentist for ordinary recall significantly less often than controls (78% vs. 93%, *p* = 0.04). In the vomiting group only 68% reported regular dentist visits which were significantly lesser than the controls (96%) (*p* = 0.016) but no difference as regard ordinary recall dentist visit was found in the no vomiting group. Other questions related to emergency dental visits, dental fear and confidence in the dental care system and preference for a male or female dentist were not statistically different between ED patients and healthy controls and neither between the vomiting and no vomiting groups (data not shown).

#### Physical exercise

ED-bad reported an average of 3.6 times weekly exercise (range 0–20), ED-good 3.2 times/week (range 0–10) and the controls 2.8 times/week but differences were not statistically significant and neither between the vomiting and no vomiting groups.

#### Conditional logistic regression

The regression analyzes showed that the ED- good consumed significantly lesser regular carbonated soft drinks (OR = 0.57) but more caffeine containing drinks (OR = 1.34) compared to controls. ED-bad had significantly lesser number of lunch meals and sweet biscuits intake per week (OR 0.59 and 0.15, respectively). When comparing ED patients in relatively good and bad disease state the former had significantly more weekly intake of lunch (OR = 1.73). Nagelkerke R^2^ for the three models ranged from 0.42 to 0.65 (Table 7).

**Table 7:** Conditional logistic regression. Final model, stepwise forward entry method

	*P*	OR	95% CI for OR
Model A: ED relatively good vs. controls (ref category: ED-good)			
Regular soft drinks	0.03	0.57	0.35–0.94
Caffeine-containing drinks	0.005	1.34	1.10–1.64
Model B: ED-bad disease state vs. controls (ref category: ED-bad)			
Lunch	0.01	0.59	0.39–0.88
Sweet biscuits	0.001	0.15	0.05–0.48
Model C: ED-good vs. bad (ref category: ED-good)			
Lunch	0.03	1.73	1.05–2.89

Included independent variables:

Model A: relatively good disease state vs. controls. Included independent variables – Consumption of Cola light, Cola regular, total carbonated regular soft drinks, total caffeine-containing drinks (L/week), apples (no/week), sweet biscuits (never/seldom or > one intake per month)

Model B: bad disease state vs. controls. Included independent variables – Cola regular, total carbonated regular soft drinks, milk (L/week), lunch (no/week), sweet biscuits, hard cheese (never/seldom or > one intake per month)

Model C: relatively good vs. bad disease state. Included independent variables – Nutrition drinks (L/week), breakfast, lunch, dinner, in-between meals (no/week), sweet biscuits (never/seldom or > one intake per month)

Model A: Nagelkerke R^2^ = 0.42. Model B: Nagelkerke R^2^ = 0.65. Model C: Nagelkerke R^2^ = 0.45

## Discussion

A common feature for ED patients is that the disease varies over the course of time with marked shifts in eating and other behaviors. In a more “active” state of the disease (presently termed “ED-bad”) an AN patient is severely restricting the calorie intake in fear of gaining weight, and an BN patient has frequent periods of severe binge eating combined with different types of compensatory behavior (e.g. self-induced vomiting, fasting, extreme exercise or use of laxative/diuretics). EDNOS patients on the other hand, may engage in any of the abnormal eating or compensatory behaviors, while not fulfilling the criteria for an AN or BN diagnosis [17]. An ED patient may also have periods during which the sign and symptoms of the disease is relatively absent and she/he is feeling pretty good (presently termed “ED-good”). It was therefore deemed important to evaluate the ED patients both in a relatively good and a bad disease state as the two conditions might present different types of eating/dietary habits and behavior. In addition to the foregoing, purging behavior is common in ED patients and one such common behavior, namely vomiting or not, was therefore further analyzed in this study.

There are a lack of well-controlled studies investigating the differences in eating habits between ED patients and healthy controls but gaining such information could be used for distinguishing patients from abnormal, but benign, eating behavior found in healthy subjects as well as phenotyping ED [18]. This study found that patients with ED has a higher intake of artificially sweetened beverages which is in agreement with previous findings [11, 13]. The preference for low-calorie dietary items is most likely connected to desire for ED patients not to gain weight in addition to that fluid intake suppress appetite [13, 19]. As regards oral health, diet drinks does not cause dental caries but is a clear risk factor for dental erosion which is a common finding in ED patients [1]. The vomiting group had a significantly higher intake of cola type light drinks which in combination with their purging behavior may substantially increase the risk for dental erosion and previous reports have found this to be true [1, 20].

Intake of caffeine-containing drinks was about double in the two ED groups compared to controls, a finding that contrast another study reporting an average intake in ED patients similar to that of the general population [21]. However, it has also been found that young girls with AN show a higher caffeine intake compared to controls which a least in part support the findings in this study [22]. Nevertheless, caffeine may suppress appetite [23] and it has been suggested that caffeine is used by ED patients to control weight and shape which is especially true for those who engage in purging or binge eating behavior [18]. There is no direct relationship between caffeine and oral health but many of the available soft drinks commonly consumed by ED patients contain caffeine and these drinks may have adverse oral consequences depending on their content of acidic/sugary constituents. The most obvious finding as regards caffeine containing drinks was in the vomiting group (ED-good) reported more than double the amount of intake caffeine compared to controls.

Meal skipping is common in ED patients and has previously been reported on [24, 25]. In this study, number of daily or weekly meals did not differ significantly between ED-good condition and controls. ED-bad on the other hand, had significantly lesser weekly intake of both breakfast, lunch and dinner compared to controls. This finding was exclusively found in the vomiting ED-bad group who skipped breakfast, lunch and dinner significantly more often than controls but no such difference was found in no vomiting patients. Within the ED group number of meals per day were reduced in bad compared to relatively good disease state. Skipping meals may have a negative effective, both on oral health [26] and a number of conditions related general health [27]. This finding highlights the importance of getting reports from ED patients not only in general terms but specifically about behavior when they are in a more active disease stage, i.e. ED-bad in this study.

Not unsurprisingly, intake of sweets and sweet biscuits were significantly lower in ED patients compared to controls and was especially pronounced during ED-bad condition. This finding was especially pronounced in the no vomiting while no differences were found in the vomiting group. One could argue therefore that the controls should have a higher risk for dental caries depending on a higher intake of sugary items. However, the no vomiting group comprised to great extent of anorectic patients (AN) or combinations thereof (EDNOS) and this group is especially vulnerable to oral diseases depending on their bad physical state including impaired salivary secretion and altered biochemical saliva composition [15].

The regression analyzes was performed with a selection of dietary items as independent variables and the final model predicted ED-good patients compared to controls on lower intake of regular soft drinks and higher intake of caffeine containing drinks. The corresponding prediction of ED-bad was lower number on lunch meals and intake of sweet biscuits. When comparing the ED-bad and good groups, the former had more frequent lunch and dinner meals and higher intake of nutrition drinks (drinks high in nutrition and energy, often recommended/prescribed to patients in special need having difficulties to eat). The results corroborate the above discussion and highlights the importance of penetrating the dietary history when examining ED patients and again the importance of getting report from their behavior in both good and bad disease state.

A common clinical impression is that ED patients more intensely and more often brush their teeth than healthy individuals. Number of brushing times per day were significantly higher among ED than in controls and more marked during ED-bad condition and especially so in the no vomiting group. These findings agree with a recently published study where more frequent toothbrushing were observed in patients with eating disorders compared to controls [28]. One study showed 32.5% of participants reported that BN patients brushed their teeth immediately after purging, [29] and another report found that tooth brushing after vomiting in ED patients had a detrimental effect on dental erosion [20]. Consequently, tooth brushing frequency should be recorded in ED patients and its tentative negative effect in relation to acidic challenges such as vomiting or soft drink intake should be informed about.

As regards awareness of dietary intake and the possible risk for oral health complications did not differ between patients and controls except that the ED groups were more aware that vomiting and brushing thereafter could damage their teeth, a finding which is positive. On the other hand, ED went less often to the dentist for regular checkups than controls which is a negative finding and patients with ED therefore needs to be encouraged to attend dental checkups more frequent since they have an increased risk for oral problems/diseases [6, 30–37]. This is deemed to be especially important for vomiting ED patients where only 68% reported regular dentist visits. Opposite finding was reported in another study where ED patients visited the dentist at least once a year, more often than controls (75% vs. 51.4%) [28]. The higher attendance of regular visits among the controls in this study (> 90%) may be explained by the well-organized Swedish public dental health system for children and adolescents which since decades back offers dental care (yearly routine checkups /emergency visits) free of charge.

The strength of the present study is that the ED group was drawn from a consecutive series of patients seeking treatment, which numbered to 65 participants during a one-year period. The relatively high participation rate of 83% (54/65) may lead to the conclusion that the results are fairly representative for ED patients seeking outpatient rehabilitation. Certain weaknesses of the study can be mentioned. The sample was small and the “risk for random occurrences” is therefore higher. The ED group was selected from an outpatient ED clinic and the results from this study may not be completely transferable to inpatients. The questionnaire was not validated according to standard procedures. However, in the construction of the questionnaire it was considered that the ED patients in many aspects present large variations not only in age and diagnoses but also regarding symptoms, expressivity, duration of their disease. The construction of the questionnaire was therefore based on available research reports in combination with the clinical experience from the multi-professional team working with this group of patients. The division of the patients into ED-good and bad was based upon the patient’s subjective opinion which is difficult to transfer an objective assessment of the real disease state. In the vomiting/no vomiting groups the response was based on a single question (“Are you presently or previously been engaged in self-induced vomiting”) and does not give any detailed information about for example frequency, timing etc. of the behavior. Such information would have been valuable to retrieve in order to more accurately analyze its consequences.

## Conclusions

The conclusions drawn from this study are that ED patients presents a number of dietary and other types of behavior that are potentially harmful for their general and oral health. For a more accurate detection of these activities, it is important that the patient report on the behaviors both when she/he is in a relatively good as well as being in a more active disease state. This could help the medical team to prescribe more adequate advice and treatment. The hypothesis that diet and other behavioral habits differ among ED patients depending on their disease state was supported.

## Supplementary information

**Additional file 1.** Questionnaires for the ED and Control Groups (in Swedish).

**Additional file 2: Table S1.** Drink Intake, Eating and Oral Hygiene Habits in Relatively Good and Bad Disease State Compared to Healthy Controls in vomiting and no vomiting patients^1^ compared to healthy controls. **Table S2.** Percentage Distribution of Intake of Dietary Items in Relatively Good and Bad Disease State in vomiting and no vomiting patients^1^ Compared to Healthy Controls.

## Data Availability

The dataset used and/or analyzed during the current study are available from the corresponding author on reasonable request.

## Abbreviations

AN: Anorexia nervosa
BN: Bulimia nervosa
ED: Eating disorder
ED-bad: self-perceived ED status reported as “bad”
ED-good: self-perceived ED status reported as “relatively good”
EDI-2: Symptom index of the eating disorder inventory-2
EDNOS: Eating disorder not otherwise specified
