# Supplementary material for: Diet and behavioral habits related to oral health in eating disorder patients: a matched case-control study
Source: J Eat Disord. 2020 Feb 27;8:7. doi: 10.1186/s40337-020-0281-z (PMC7045484; doi:10.1186/s40337-020-0281-z)
Supplement: Supplementary file 1 — Additional file 1. Questionnaires for the ED and Control Groups (in Swedish). [file 40337_2020_281_MOESM1_ESM.zip › Supplementary file - Questionnaire_ ED group.pdf]

1. **Röker du**
   a. Nej, har aldrig rökt
   b. Ja, jag röker dagligen
   c. Ja, jag röker ibland
   d. Tidigare, men inte nu

2. **Om du röker/rökt: Hur mycket och hur länge**
   a. Jag har aldrig rökt
   b. ……..cigaretter/dag under ….…………...år

3. **Snusar du**
   a. Nej, jag har aldrig snusat
   b. Ja, jag snusar
   c. Ja, jag snusar ibland
   d. Tidigare, men inte nu

4. **Om du snusar/snusat: Hur mycket och hur länge**
   a. Jag har aldrig snusat
   b. …….. …dosa/dosor per dag under …..… …år

5. **Besöker du tandvården regelbundet**
   a. Ja
   b. Bara om jag har besvär
   c. Nej, jag besöker <u>inte</u> tandvården regelbundet

6. **Mitt senaste besök hos**
   a. Tandläkare var för …….. mån/år sedan
   b. Tandhygienist var för …….. mån/år sedan

7. **Har du besökt tandvården <u>akut</u> de sista två åren**
   a. Ja, ……. gång/gånger
   b. Nej

8. **Har du undersökts av tandvården de senaste två åren (***annat än ev. akutbesök)*
   Kryssa det mest lämpliga
   a. Ja
   b. Nej, det har inte blivit av
   c. Nej, jag skulle velat men har inte haft råd
   d. Nej, tycker inte att jag haft behov
   e. Annan orsak…………………………….

9. **Kontaktar du då tandvården om du har tandbesvär**
   a. Har inte haft tandbesvär
   b. Alltid
   c. Ofta
   d. Ibland
   e. Sällan /Aldrig

10. **Om du inte sökt tandvård trots besvär: Varför har du inte gjort det**
    Ringa in ett eller flera alternativ
    a. Har inte haft besvär
    b. Har inte haft tid själv
    c. Besvären försvann
    d. Min ätstörning tar mycket tid
    e. Har inte haft råd
    f. Kändes inte viktigt
    g. Har inte blivit av
    h. Jag undviker tandvård
    i. Är rädd

11. **Upplever du dig själv som tandvårdsrädd**
    a. Ja, lite
    b. Ja, mycket
    c. Nej

12. **Var du tandvårdsrädd som barn**
    a. Ja
    b. Nej

13. **Om du är tandvårdsrädd: Varför?**
    Ringa in ett eller flera alternativ
    a. Jag är inte tandvårdsrädd
    b. Jag är rädd för att det ska göra ont
    c. Jag är rädd för sprutor
    d. Jag är rädd för att tandläkaren ska hitta något fel
    e. Jag är rädd för att tandläkaren ska se att jag har en ätstörning
    f. Jag är rädd för att det ska kosta pengar
    g. Jag vet inte vad som ska hända
    h. Annat:……………………………………………

14. **Tycker du att tandvården kunnat ge dig den hjälp du behöver**
    a. Har inte sökt och inte haft behov av hjälp
    b. Har trots tandbesvär inte sökt hjälp
    c. Ja, jag har fått den hjälp jag behöver
    d. Ja, men jag har inte haft råd att betala undersökning
    e. Ja, men jag har inte haft råd med föreslagen tandvård
    f. Nej, jag har inte fått den hjälp jag behöver
    g. Tandvården har <u>inte</u> kunnat erbjuda tider
    h. Annat:……………………………………

15. **Är du under tandbehandling nu**
    a. Ja
    b. Nej, men jag väntar på att bli kallad
    c. Nej

16. **Har du tandbesvär nu**
    a. Ja mycket
    b. Ja, lite
    c. Nej

17. **Om du undviker tandvård eller bara besöker tandvården om du har ont: <u>Varför</u> gör du det**
    Ringa in ett eller flera alternativ
    a. Jag undviker inte tandvård
    b. Jag är rädd för att det ska göra ont
    c. Jag är rädd för att tandläkaren ska hitta något fel
    d. Jag är rädd för att tandläkaren ska se att jag har en ätstörning
    e. Jag är rädd för att det ska kosta pengar
    f. Jag vet inte vad som ska hända
    g. Annat:……………………………………………

18. **Hur har du löst dina tandvårdskostnader**
    Ringa in ett eller flera alternativ
    a. Har inte betalat något då jag är under 20 år
    b. Har inte fått någon tandvård
    c. Har betalat själv
    d. Har lånat pengar
    e. Någon anhörig eller mina föräldrar har betalat
    f. Socialförvaltningen har betalat
    g. Försäkringskassan har givit mig <u>extra</u> stöd

19. När fick du din ätstörning första gången
a. För ……………..månader/år sedan
b. Annat:………………………………………

20. Föredrar du manlig eller kvinnlig tandläkare
a. Jag föredrar en kvinnlig
b. Jag föredrar en manlig
c. Det spelar ingen roll

21. Hur upplever du din egen tandhälsa
a. Bra
b. Ganska bra
c. Inte så bra
d. Dåligt
e. Mycket dåligt

22. Tycker du att Dina tänder ändrat sig efter det att du fått en ätstörning. *(Gäller ej för dig som just växlat mjölktänderna). Ringa in ett eller flera alternativ*
a. Jag tycker att jag har bättre tänder nu
b. Jag tycker inte att det är någon skillnad
c. Mina tänder är mer slitna nu
d. Jag tycker mina tänder blivit vassa
e. Jag tycker mina tänder blivit gulare
f. Bettet har ändrat sig
g. Mer hål i tänderna
h. Mer tandköttsblödning
i. Annat………………………………………………

23. Tycker du dina tänder är slitna
a. Ja, mycket slitna
b. Ja, ganska slitna
c. Ja, lite slitna
d. Nej, inte alls

24. Tror du att du behöver behandling för slitna tänder
a. Ja
b. Nej
c. Vet ej
d. Jag har redan fått behandling för slitna tänder

25. Har du haft tandställning
a. Nej
b. Nej, men jag borde ha haft det
c. Ja, men jag slutförde inte behandlingen
d. Ja

26. Vem gjorde dig först medveten om att du har en ätstörning
a. Jag förstod själv
b. Förälder
c. En anhörig/vän
d. Tandvården
e. Sjukvården
f. Skol-studenthälsovården
g. Övrigt:…..…………………………………………

27. Vem tog en första kontakt med Eriksbergsgården
a. Jag själv
b. Förälder
c. En anhörig/vän
d. Tandvården
e. Sjukvården
f. Skol-studenthälsovården
g. Annat……………………………………..

28. Om dina tänder isar/smärtar i vilket/vilka sammanhang sker detta
Ringa in ett eller flera alternativ
a. Mina tänder isar eller smärtar sällan /aldrig
b. Alltid, även då jag inget gör
c. När jag äter sött /surt/ det mesta
d. När jag tuggar
e. När jag dricker kallt
f. När jag dricker varmt
g. Vid tandborstning
h. Vid kräkning
i. På morgonen
j. På dagen
k. På natten
l. Om jag är ute i kyla

29. Vem upptäckte dina tandproblem
a. Jag har inga tandproblem
b. Jag själv
c. Tandvården
d. Någon annan ange vem:…………………………

30. Vet din tandläkare att du har en ätstörning
Ringa in ett eller flera alternativ
a. Ja, jag har själv berättat det
b. Ja, tandläkaren frågade och jag sa att det stämde
c. Nej, tandläkaren frågade men jag sa att det inte stämde
d. Nej, jag har varit där men tandläkaren har inte sagt något
e. Nej, jag tycker inte att jag behöver berätta det
f. Jag vet att jag borde berätta men har inte gjort det
g. Jag har inte varit hos tandläkaren

31. Har du fått information om hur dina tänder kan påverkas av en ätstörning
Ringa in ett eller flera alternativ
a. Nej, jag har inte fått någon information
b. Ja, från min tandläkare
c. Ja, från min tandhygienist
d. Ja, från personal på ätstörningsmottagningen
e. Ja, jag har fått information från tidningar och TV
f. Ja, jag har fått information från annat håll, ange från var: ……………………………………………..
……………………..................................…….

32. Hur många gånger i veckan brukar du träna fysiskt
a. när du mår ganska bra i din ätstörning
   i. ……………gånger/v/mån
b. när du mår dåligt i din ätstörning
   i. ………………gånger/v/mån

Ringa in ett alternativ	Sällan eller Aldrig	En till flera ggr per månad	En till flera ggr per vecka	Varje dag, av och till	Varje dag, hela tiden
**33.** Har du besvär av huvudvärk	1	2	3	4	5
**34.** Känner du dig tung i huvudet	1	2	3	4	5
**35.** Har du smärta eller värk i ansikte och/eller käkar	1	2	3	4	5
**36.** Gör det ont när du rör underkäken och tuggar	1	2	3	4	5
**37.** Har du en klump i halsen	1	2	3	4	5
**38.** Har du svårt att gapa/bita över en stor tugga	1	2	3	4	5
**39.** Är du trött i käkarna	1	2	3	4	5
**40.** Knäpper det från käkarna	1	2	3	4	5
**41.** Har du skrapljud från käkarna	1	2	3	4	5
**42.** Hoppar underkäken ur led och/eller låser sig	1	2	3	4	5
**43.** Gnider, pressar eller gnisslar du tänder under dagen	1	2	3	4	5
**44.** Gnider, pressar eller gnisslar du tänder under natten	1	2	3	4	5
**45.** Pressar du tungan mot tänderna/ gommen under dagen	1	2	3	4	5
**46.** Pressar du tungan mot tänderna/ gommen under natten	1	2	3	4	5
**47.** Har du svårt att tugga	1	2	3	4	5
**48.** Tuggar du tuggummi	1	2	3	4	5
**49.** Är du yr i huvudet	1	2	3	4	5
**50.** Har du svårt att koncentrera dig	1	2	3	4	5
**51.** Har du susningar eller ringningar i öronen	1	2	3	4	5
**52.** Har du svårt att sova	1	2	3	4	5
**53.** Är du spänd i käkarna när du vaknar på morgonen	1	2	3	4	5
**54.** Snarkar du	1	2	3	4	5
**55.** Har du problem från dina tänder	1	2	3	4	5
**56.** Hur ofta isar/smärtar dina tänder när du äter	1	2	3	4	5
**57.** Hur ofta isar/smärtar dina tänder om du dricker kallt	1	2	3	4	5
**58.** Hur ofta isar/smärtar dina tänder om du dricker varmt	1	2	3	4	5
**59.** Besväras du av kväljningar	1	2	3	4	5
**60.** Tycker du själv att du är torr i munnen	1	2	3	4	5
**61.** Smakar det illa i din mun	1	2	3	4	5
**62.** Tror du att du luktar illa ur munnen	1	2	3	4	5
**63.** Har du beläggningar på tungan	1	2	3	4	5
**64.** Svider eller bränner det i munnen	1	2	3	4	5
**65.** Svider eller bränner det på tungan	1	2	3	4	5
**66.** Brukar du ha blåsor i munnen	1	2	3	4	5
**67.** Har du sår i munnen	1	2	3	4	5
**68.** Har du sår i mungiporna	1	2	3	4	5
**69.** Blöder ditt tandkött	1	2	3	4	5
**70.** Har tandbesvär <u>påverkat</u> vad du äter och dricker det senaste året	1	2	3	4	5
**71.** Har tandbesvär <u>hindrat</u> dig från att äta/dricka det senaste året	1	2	3	4	5

72. Använder du bettskena
a. Ja
b. Tidigare men inte nu
c. Nej

73. Om du använt/använder bettskena varför har du i så fall gjort det *Ringa in ett eller flera alternativ*
a. Jag har inte använt bettskena
b. Mina tänder var/är slitna
c. Gnisslar eller pressar tänder
d. Pga. värk i huvud ansikte eller käkar
e. Ont i munnen eller tungan
f. Annat…………………………………………

74. Besväras du av kväljningar
a. Ja
b. Tidigare men inte nu
c. Nej

75. Om du besväras av kväljningar: när besväras du *Ringa in ett eller flera alternativ*
a. Jag besväras inte av kväljningar
b. Vid tandborstning
c. När jag äter
d. När jag har ätit
e. När jag dricker
f. När jag har druckit
g. Vid vissa lukter
h. Speciell mat
i. I samband med tandvård
j. När jag mår dåligt i min ätstörning
k. Annat:…………………………………………

Hur andas du på:

	**Genom**			
	**Munnen**	**Näsan**	**Mun och näsa**	**Vet ej**
**76.** Dagen när du är vaken				
**77.** Natten när du sover				

Hur ofta, hur länge, med vilken tandkräm och med hur mycket tandkräm borstar du tänderna i perioder:

	Ant. gånger (per dygn)	Hur länge (min)	Tandkräm (namn)	Centimeter Tandkräm
**78.** När Du mår ganska bra i din ätstörning				
**79.** När du mår dåligt i din ätstörning				

Vilken borstmetod använder du vid tandborstning när:

	Ingen speciell	Upp-och-ned (vertikalt)	Fram-och-tillbaka (horisontellt)	Kombination av Upp-och-ned & Fram-och-tillbaka
**80.** Du mår ganska bra i din ätstörning				
**81.** Mår dåligt i din ätstörning				

När borstar du dina tänder när:

Kryssa för ett eller flera alternativ	Borstar inte tänderna	Morgon	Kväll	Natt	Före kräkning	Under kräkning	Efter kräkning	Före måltid	Efter måltid
**82.** Du mår ganska bra i din ätstörning:									
**83.** Mår dåligt i din ätstörning									

84. Sköljer du munnen med något efter tandborstning (munvatten etc.)
a. Nej
b. Ja med……………………………………….

85. Vilken typ av tandborste använder du
a. Vanlig tandborste
b. El-tandborste

86. Rengör du mellan tänderna
Ringa in ett eller flera alternativ
a. Nej, inte alls
b. Ja, med tandtråd
c. Ja, med tandsticka
d. Ja med mellanrumsborste
e. Annat………………………………………..

87. Hur ofta rengör du mellan tänderna
a. Jag rengör inte mellan tänderna
b. Dagligen
c. Några gånger i veckan
d. Någon gång i veckan
e. Varje månad eller mer sällan

88. Hur ofta använder du fluor förutom den fluor som finns i tandkräm
a. Inte alls
b. Dagligen
c. Flera gånger dagligen
d. Varje vecka
e. Flera gånger i veckan
f. Varje månad

89. Om du använder fluor:
Vilket/vilka fluorpreparat använder du då
Ringa in ett eller flera alternativ
a. Nej, jag använder inte fluor
b. Ja, tandkräm med fluor
c. Ja, fluorsköljning
d. Ja, fluortabletter
e. Ja, fluortuggummi
f. Ja, fluor i speciella skedar

90. Om du använder fluor:
Vem har rekommenderat dig att använda fluor
a. Jag använder inte fluor
b. Tandläkare
c. Tandhygienist
d. Sjukvårdspersonal
e. Media
f. Annat………………………………………………
…………………………………………

91. Använder du salivstimulerande medel
a. Nej
b. Ja, vilket typ……………………………………….

92. Hur ofta använder du salivstimulerande medel
a. Jag använder inte salivstimulerande
b. Dagligen
c. Varje vecka
d. Någon gång i månaden/aldrig

93. Om du använder salivstimulerande:
Vem har rekommenderat dig att använda detta
a. Jag använder inte salivstimulerande
b. Tandläkare
c. Tandhygienist
d. Sjukvårdspersonal
e. Media
f. Annat………………………………………………
…………………………………………

94. Hur känns ytan på dina tänder
a. Inget speciellt
b. Glatt
c. Sträv
d. Trasiga
e. Annat:…………………………………………

95. Om du svullnar nedanför örat: Hur ofta och hur mycket *(dvs. svullnad i den körteln man kan få påssjuka i)*
a. Jag svullnar inte upp nedanför örat
b. Hur ofta ………………………………………
c. Hur länge……………………………………

96. Är det båda sidor som svullnar nedanför örat
a. Ingen sida svullnar upp
b. Bara ena sidan
c. Båda sidor
d. Ibland en ibland båda sidor

97. Hur upplever du att det svullnar nedanför örat
a. Det svullnar inte upp
b. Jag bryr mig inte om det
c. Jag tycker det känns klumpigt
d. Jag tycker det är fult
e. Jag blir orolig för att det kanske kan vara farligt
f. Annat………………………………………………
…………………………………………

98. Om du svullnar upp under hakan: Hur ofta och hur mycket svullnar det under hakan
a. Jag svullnar inte upp under hakan
b. Hur ofta ………………………………………
c. Hur länge……………………………………

99. Hur upplever du att det svullnar under hakan
a. Det svullnar inte upp
b. Jag bryr mig inte om det
c. Jag tycker det känns klumpigt
d. Jag tycker det är fult
e. Jag blir orolig för att det kanske kan vara farligt
f. Annat………………………………………………
…………………………………………

100. Är det båda sidor som svullnar under hakan
a. Ingen sida svullnar upp
b. Bara ena sidan
c. Båda sidor
d. Ibland en ibland båda sidor

Hur mycket och vad brukar du dricka

	När du mår				Anm.
**Dryck**	**ganska bra i din ätstörning**		**dåligt i din ätstörning**		**Anm.**
	**ml**	**Hur ofta/dag/v**	**ml**	**Hur ofta/dag/v**	
**Cola-drycker:**					
**101.** Light					
**102.** Med socker					
**Annan läsk:**					
**103.** Light					
**104.** Med socker					
**105.** Sportdryck:					
**106.** Äppelcidervinäger:					
**Juice:**					
**107.** Typ					
**Näringsdryck:**					
**108.** Typ					
**Te:**					
**109.** Typ av te:					
**110.** Utan socker					
**111.** Med socker					
**Kaffe:**					
**112.** Utan socker					
**113.** Med socker					
**114.** Mjölk					
**115.** C-vitamin brus					
**116.** Vitamin brus					
**117.** Vatten					
**118. Frukt**	**Ant./dag/v**		**Ant./dag/v**		**Anm.**
**119.** Äpple					
**120.** Päron					
**121.** Apelsin					
**122.** Citron					**Suger ja □ nej □**
**123.** Mandarin					
**124.** Grape					
**125.** Banan					
**126. Annat:**					

127. Har du tidigare, mer än 1 år tillbaka, ätit/druckit mycket av ovanstående
Ange vad och hur mycket……………………………………………………………………………………
………..
……….

	Ja	Nej
**128.** Tror du att vanlig läsk (med socker) kan skada dina tänder		
**129.** Tror du att light läsk (utan vanligt socker) kan skada dina tänder		
**130.** Tror du att fruktjuice kan skada dina tänder		
**131.** Tror du att sportdryck kan skada dina tänder		
**132.** Tror du att frukt kan skada dina tänder		
**133.** Tror du att godis kan skada dina tänder		
**134.** Har du fått särskild förebyggande tandbehandling/råd pga. av din ätstörning		
**135.** Tycker du att du fått mycket tandbehandling jämfört andra i din ålder		
**136.** Har du blekt dina tänder		
**137.** Har du haft perioder på minst 6 månader då du varit utan symptom från din sjukdom:		
**138.** Har du besökt tandvården sedan du fick din ätstörning		
**139.** Behöver du behandling för isningar/smärta i tänderna		
**140.** Har du förtroende för tandvården		
**141.** Tror du att tandborstning efter att du druckit juice kan skada dina tänder		
**142.** Tror du att tandborstning efter att du druckit light läsk kan skada dina tänder		
**143.** Tror du att tandläkaren kan se i munnen att du har en ätstörning		
**144.** Tror du att det kan vara dåligt för munnen att inte äta/dricka		
**145.** Kräkning kan skada dina tänder		
**146.** Tandborstning efter kräkning kan skada dina tänder		

När du mår ganska bra i din ätstörning, vad dricker du till:

**Vad dricker du till**	**Produkt**	**Hur mycket (ml)**
**147.** Frukost		
**148.** Lunch		
**149.** Middag		
**150.** Kvällen		
**151.** Under natten		
**152.** Mellan måltiderna		
**153.** Under träning		
**154.** Efter träning		
**155.** Hur många mål mat äter du /dag		

När du mår ganska bra i din ätstörning:

**Hur ofta äter du**	Sällan eller Aldrig	En till flera ggr per månad	En till flera ggr per vecka	Dagligen	Flera ggr dagligen
**156.** Godis	1	2	3	4	5
**157.** Kakor/bullar/kex	1	2	3	4	5
**158.** Hård ost	1	2	3	4	5
**159.** Filmjölk/yoghurt	1	2	3	4	5

160. När du mår ganska bra i din ätstörning: Hur ofta äter du

a. Frukost ………………….gånger/vecka
b. Lunch……………………gånger/vecka
c. Middag…………………..gånger/vecka
d. Mellanmål……………….gånger/vecka

När du mår dåligt i din ätstörning, vad dricker du till:

**Vad dricker du till**	**Produkt**	**Hur mycket (ml)**
**161.** Frukost		
**162.** Lunch		
**163.** Middag		
**164.** Kvällen		
**165.** Under natten		
**166.** Mellan måltiderna		
**167.** Under träning		
**168.** Hur många mål mat äter du /dag		

När du mår dåligt i din ätstörning:

**Hur ofta äter du**	Sällan eller Aldrig	En till flera ggr per månad	En till flera ggr per vecka	Varje dag, av och till	Varje dag, hela tiden
**169.** Godis	1	2	3	4	5
**170.** Kakor/bullar/kex	1	2	3	4	5
**171.** Hård ost	1	2	3	4	5
**172.** Filmjölk/yoghurt	1	2	3	4	5

173. När du mår dåligt i din ätstörning: Hur ofta äter du
a. Frukost ………………….gånger/vecka
b. Lunch……………………gånger/vecka
c. Middag…………………..gånger/vecka
d. Mellanmål……………….gånger/vecka

174. Om du är vegetarian, vilken typ och hur länge har du varit det
a. Jag är inte vegetarian
b. Ja, jag är ……………………
sedan…………………………….år/månader
c. Jag har varit vegetarian tidigare
Typ……………………………………..
Tid………………………………år/månader

175. Framkallar du kräkningar själv
a. Ja
b. Nej

176. Har du tidigare framkallat kräkningar själv
a. Ja
b. Nej

177. Brukar du ibland helt förlora kontrollen över vad och hur mycket du äter
a. Ja
b. Nej

178. Om du förlorar kontrollen över vad och hur mycket du äter
a. Jag förlorar inte kontrollen
b. Vad är det du oftast äter då
………………………………………………………
c. Vad är det du oftast dricker då
………………………………………………………

179. Om du förlorar kontrollen över vad och hur mycket du äter: Ungefär hur lång tid ”per gång” gör du det
a. Jag förlorar inte kontrollen
b. Ungefär…………min……………timmar

180. Ungefär under hur lång ”tidsperiod” har det hänt att du förlorat kontrollen över vad och hur mycket du äter
a. Antal år ……månader……………………...

181. Vad gör du efter det att du ätit okontrollerat
a. Jag äter inte okontrollerat
b. ……………………………………………

182. Under hur lång tidsperiod har det förekommit att du själv framkallat kräkning
……………………………………………………
…………………………………………….

183. Hur många gånger om dygnet har du kräkts det senaste halvåret
a. *……gånger per dygn………per vecka… per månad…………………………………………*

184. Hur många gånger om dygnet har du kräkts när du mår som sämst
a. *……gånger per dygn…per vecka….per månad*

185. Hur många gånger om dygnet borstar du tänderna i perioder av kräkning
a. *……gånger per dygn…per vecka….per månad*
……………………………………………………......

186. Hur lång tid efter det att du ätit brukar du <u>oftast</u> kräkas
a. Inom 15 min
b. Inom 30 min
c. Inom en timme
d. Inom två timmar
e. Inom tre timmar
f. Efter mer än tre timmar
g. Annat………………………………………

187. Hur framkallar du kräkning
a. Fingrar i halsen
b. Kan göra med magen så att jag kräks
c. Vet ej - mår bara illa och kräks
d. Annat
……………………………………………….
……………………………………………….

188. Hur många gånger kräks du om du förlorat kontrollen över vad/hur mycket du äter
………………………………………………………

189. Hur känns det i munnen efter kräkning
………………………………………………………
………………………………………………………

190. Vad gör du med munnen efter en kräkning
Ringa in ett eller flera alternativ
a. Sköljer med vatten
b. Sköljer med fluor
c. Dricker Cola
d. Tar en tablett
Med socker ☐ Utan socker ☐
e. Borstar tungan
f. Borstar tänderna
g. Jag gör ingenting
h. Dricker kaffe
i. Annat:……………………………………
……………………………………………...

191. Om du sköljer i samband med kräkning: <u>Vad</u> är det du sköljer
a. Jag sköljer inte
b. Munnen
c. Magen
d. Munnen och magen
e. Annat………………………………………
……………………………………………….

192. Om du sköljer: <u>Med vad</u> sköljer du oftast
a. Jag sköljer inte
b. Vatten

193. Hur mycket vätska/dricka sköljer du med
a. Ungefär:……………………………………
……………………………………………..

194. Om du sköljer i samband med kräkning: <u>Varför</u> gör du det
a. ……………………………………………
……………………………………………

195. Om du borstar tänderna efter kräkning varför gör du detta
a. Jag borstar inte tänderna efter kräkning
b. För att få bättre smak i munnen
c. För att mina tänder <u>inte</u> ska bli förstörda
d. För att jag <u>inte</u> ska lukta illa ur munnen
Annat…………………………………………
…………………………………………………..

196. Om du borstar tänderna efter kräkning med vilken och med hur mycket tandkräm borstar du då

Hur länge (min)	Tandkräm (namn)	Centimeter Tandkräm
