# Supplementary material for: Diet and behavioral habits related to oral health in eating disorder patients: a matched case-control study
Source: J Eat Disord. 2020 Feb 27;8:7. doi: 10.1186/s40337-020-0281-z (PMC7045484; doi:10.1186/s40337-020-0281-z)
Supplement: Supplementary file 1 — Additional file 1. Questionnaires for the ED and Control Groups (in Swedish). [file 40337_2020_281_MOESM1_ESM.zip › Supplementary file - Questionnaire_Control group.pdf]

1. **Röker du**
   a. Nej, har aldrig rökt
   b. Ja, jag röker dagligen
   c. Ja, jag röker ibland
   d. Tidigare, men inte nu

2. **Om du röker/rökt: Hur mycket och hur länge**
   a. Jag har aldrig rökt
   b. ……..cigaretter/dag under ….…………...år

3. **Snusar du**
   a. Nej, jag har aldrig snusat
   b. Ja, jag snusar
   c. Ja, jag snusar ibland
   d. Tidigare, men inte nu

4. **Om du snusar/snusat: Hur mycket och hur länge**
   a. Jag har aldrig snusat
   b. …….. …dosa/dosor per dag under …..… …år

5. **Besöker du tandvården regelbundet**
   a. Ja
   b. Bara om jag har besvär
   c. Nej, jag besöker inte tandvården regelbundet

6. **Mitt senaste besök hos**
   a. Tandläkare var för …….. mån/år sedan
   b. Tandhygienist var för …….. mån/år sedan

7. **Har du besökt tandvården akut de sista två åren**
   a. Ja, ……. gång/gånger
   b. Nej

8. **Har du undersökts av tandvården de senaste två åren (***annat än ev. akutbesök)*
   Kryssa det mest lämpliga
   a. Ja
   b. Nej, det har inte blivit av
   c. Nej, jag skulle velat men har inte haft råd
   d. Nej, tycker inte att jag haft behov
   e. Annan orsak…………………………….

9. **Kontaktar du då tandvården om du har tandbesvär**
   a. Har inte haft tandbesvär
   b. Alltid
   c. Ofta
   d. Ibland
   e. Sällan /Aldrig

10. **Om du inte sökt tandvård trots besvär: Varför har du inte gjort det**
   Ringa in ett eller flera alternativ
   a. Har inte haft besvär
   b. Har inte haft tid själv
   c. Besvären försvann
   e. Har inte haft råd
   f. Kändes inte viktigt
   g. Har inte blivit av
   h. Jag undviker tandvård
   i. Är rädd

11. **Upplever du dig själv som tandvårdsrädd**
   a. Ja, lite
   b. Ja, mycket
   c. Nej

12. **Var du tandvårdsrädd som barn**
   a. Ja
   b. Nej

13. **Om du är tandvårdsrädd: Varför?**
   Ringa in ett eller flera alternativ
   a. Jag är inte tandvårdsrädd
   b. Jag är rädd för att det ska göra ont
   c. Jag är rädd för sprutor
   d. Jag är rädd för att tandläkaren ska hitta något fel
   f. Jag är rädd för att det ska kosta pengar
   g. Jag vet inte vad som ska hända
   h. Annat:………………………………………………

14. **Tycker du att tandvården kunnat ge dig den hjälp du behöver**
   a. Har inte sökt och inte haft behov av hjälp
   b. Har trots tandbesvär inte sökt hjälp
   c. Ja, jag har fått den hjälp jag behöver
   d. Ja, men jag har inte haft råd att betala undersökning
   e. Ja, men jag har inte haft råd med föreslagen tandvård
   f. Nej, jag har inte fått den hjälp jag behöver
   g. Tandvården har inte kunnat erbjuda tider
   h. Annat:…………………………………….

15. **Är du under tandbehandling nu**
   a. Ja
   b. Nej, men jag väntar på att bli kallad
   c. Nej

16. **Har du tandbesvär nu**
   a. Ja mycket
   b. Ja, lite
   c. Nej

17. **Om du undviker tandvård eller bara besöker tandvården om du har ont: Varför gör du det**
   Ringa in ett eller flera alternativ
   a. Jag undviker inte tandvård
   b. Jag är rädd för att det ska göra ont
   c. Jag är rädd för att tandläkaren ska hitta något fel
   e. Jag är rädd för att det ska kosta pengar
   f. Jag vet inte vad som ska hända
   g. Annat:………………………………………………

18. **Hur har du löst dina tandvårdskostnader**
   Ringa in ett eller flera alternativ
   a. Har inte betalat något då jag är under 20 år
   b. Har inte fått någon tandvård
   c. Har betalat själv
   d. Har lånat pengar
   e. Någon anhörig eller mina föräldrar har betalat
   f. Socialförvaltningen har betalat
   g. Försäkringskassan har givit mig extra stöd

20. Föredrar du manlig eller kvinnlig tandläkare
a. Jag föredrar en kvinnlig
b. Jag föredrar en manlig
c. Det spelar ingen roll

21. Hur upplever du din egen tandhälsa
a. Bra
b. Ganska bra
c. Inte så bra
d. Dåligt
e. Mycket dåligt

23. Tycker du dina tänder är slitna
a. Ja, mycket slitna
b. Ja, ganska slitna
c. Ja, lite slitna
d. Nej, inte alls

24. Tror du att du behöver behandling för slitna tänder
a. Ja
b. Nej
c. Vet ej
d. Jag har redan fått behandling för slitna tänder

25. Har du haft tandställning
a. Nej
b. Nej, men jag borde ha haft det
c. Ja, men jag slutförde inte behandlingen
d. Ja

28. Om dina tänder isar/smärtar i vilket/vilka sammanhang sker detta
Ringa in ett eller flera alternativ
a. Mina tänder isar eller smärtar sällan /aldrig
b. Alltid, även då jag inget gör
c. När jag äter sött /surt/ det mesta
d. När jag tuggar
e. När jag dricker kallt
f. När jag dricker varmt
g. Vid tandborstning
i. På morgonen
j. På dagen
k. På natten
l. Om jag är ute i kyla

29. Vem upptäckte dina tandproblem
a. Jag har inga tandproblem
b. Jag själv
c. Tandvården
d. Någon annan ange vem:…………………………

32. Hur många gånger i veckan brukar du träna fysiskt
………………gånger/v/mån

Ringa in ett alternativ	Sällan eller aldrig	En till flera ggr per månad	En till flera ggr per vecka	Varje dag, av och till	Varje dag, hela tiden
**33.** Har du besvär av huvudvärk	1	2	3	4	5
**34.** Känner du dig tung i huvudet	1	2	3	4	5
**35.** Har du smärta eller värk i ansikte och/eller käkar	1	2	3	4	5
**36.** Gör det ont när du rör underkäken och tuggar	1	2	3	4	5
**37.** Har du en klump i halsen	1	2	3	4	5
**38.** Har du svårt att gapa/bita över en stor tugga	1	2	3	4	5
**39.** Är du trött i käkarna	1	2	3	4	5
**40.** Knäpper det från käkarna	1	2	3	4	5
**41.** Har du skrapljud från käkarna	1	2	3	4	5
**42.** Hoppar underkäken ur led och/eller låser sig	1	2	3	4	5
**43.** Gnider, pressar eller gnisslar du tänder under dagen	1	2	3	4	5
**44.** Gnider, pressar eller gnisslar du tänder under natten	1	2	3	4	5
**45.** Pressar du tungan mot tänderna/ gommen under dagen	1	2	3	4	5
**46.** Pressar du tungan mot tänderna/ gommen under natten	1	2	3	4	5
**47.** Har du svårt att tugga	1	2	3	4	5
**48.** Tuggar du tuggummi	1	2	3	4	5
**49.** Är du yr i huvudet	1	2	3	4	5
**50.** Har du svårt att koncentrera dig	1	2	3	4	5
**51.** Har du susningar eller ringningar i öronen	1	2	3	4	5
**52.** Har du svårt att sova	1	2	3	4	5
**53.** Är du spänd i käkarna när du vaknar på morgonen	1	2	3	4	5
**54.** Snarkar du	1	2	3	4	5
**55.** Har du problem från dina tänder	1	2	3	4	5
**56.** Hur ofta isar/smärtar dina tänder när du äter	1	2	3	4	5
**57.** Hur ofta isar/smärtar dina tänder om du dricker kallt	1	2	3	4	5
**58.** Hur ofta isar/smärtar dina tänder om du dricker varmt	1	2	3	4	5
**59.** Besväras du av kväljningar	1	2	3	4	5
**60.** Tycker du själv att du är torr i munnen	1	2	3	4	5
**61.** Smakar det illa i din mun	1	2	3	4	5
**62.** Tror du att du luktar illa ur munnen	1	2	3	4	5
**63.** Har du beläggningar på tungan	1	2	3	4	5
**64.** Svider eller bränner det i munnen	1	2	3	4	5
**65.** Svider eller bränner det på tungan	1	2	3	4	5
**66.** Brukar du ha blåsor i munnen	1	2	3	4	5
**67.** Har du sår i munnen	1	2	3	4	5
**68.** Har du sår i mungiporna	1	2	3	4	5
**69.** Blöder ditt tandkött	1	2	3	4	5
**70.** Har tandbesvär påverkat vad du äter och dricker det senaste året	1	2	3	4	5
**71.** Har tandbesvär hindrat dig från att äta/dricka det senaste året	1	2	3	4	5

72. Använder du bettskena
a. Ja
b. Tidigare men inte nu
c. Nej

73. Om du använt/använder bettskena varför har du i så fall gjort det *Ringa in ett eller flera alternativ*
a. Jag har inte använt bettskena
b. Mina tänder var/är slitna
c. Gnisslar eller pressar tänder
d. Pga. värk i huvud ansikte eller käkar
e. Ont i munnen eller tungan
f. Annat…………………………………………

74. Besväras du av kväljningar
a. Ja
b. Tidigare men inte nu
c. Nej

75. Om du besväras av kväljningar: när besväras du
Ringa in ett eller flera alternativ
a. Jag besväras inte av kväljningar
b. Vid tandborstning
c. När jag äter
d. När jag har ätit
e. När jag dricker
f. När jag har druckit
g. Vid vissa lukter
h. Speciell mat
i. I samband med tandvård
k. Annat:…………………………………………

751. Har du problem med magen
a. Ja
b. Nej
Om ja, vad?…………………………………………..
…………………………………………………….
…………………………………………………….
Hur länge?………………….. (månader / år)

Hur andas du:

	**Genom**			
	**Munnen**	**Näsan**	**Mun och näsa**	**Vet ej**
**76.** på dagen när du är vaken				
**77.** på natten när du sover				

Hur ofta, hur länge, med vilken tandkräm och med hur mycket tandkräm borstar du tänderna:

	Ant. gånger (per dygn)	Hur länge (min)	Tandkräm (namn)	Centimeter Tandkräm
**78.**				

Vilken borstmetod använder du vid tandborstning:

	Ingen speciell	Upp-och-ned (vertikalt)	Fram-och-tillbaka (horisontellt)	Kombination av Upp-och-ned & Fram-och-tillbaka
**80.**				

När borstar du dina tänder:

Kryssa för ett eller flera alternativ	Borstar inte tänderna	Morgon	Kväll	Natt	Före måltid	Efter måltid
**82.**						

84. Sköljer du munnen med något efter tandborstning (munvatten etc.)
a. Nej
b. Ja med……………………………………….

85. Vilken typ av tandborste använder du
a. Vanlig tandborste
b. El-tandborste

86. Rengör du mellan tänderna
Ringa in ett eller flera alternativ
a. Nej, inte alls
b. Ja, med tandtråd
c. Ja, med tandsticka
d. Ja med mellanrumsborste
e. Annat………………………………………..

87. Hur ofta rengör du mellan tänderna
a. Jag rengör inte mellan tänderna
b. Dagligen
c. Några gånger i veckan
d. Någon gång i veckan
e. Varje månad eller mer sällan

88. Hur ofta använder du fluor förutom den fluor som finns i tandkräm
a. Inte alls
b. Dagligen
c. Flera gånger dagligen
d. Varje vecka
e. Flera gånger i veckan
f. Varje månad

89. Om du använder fluor:
Vilket/vilka fluorpreparat använder du då
Ringa in ett eller flera alternativ
a. Nej, jag använder inte fluor
b. Ja, tandkräm med fluor
c. Ja, fluorsköljning
d. Ja, fluortabletter
e. Ja, fluortuggummi
f. Ja, fluor i speciella skedar

90. Om du använder fluor:
Vem har rekommenderat dig att använda fluor
a. Jag använder inte fluor
b. Tandläkare
c. Tandhygienist
d. Sjukvårdspersonal
e. Media
f. Annat………………………………………………
…………………………………………

91. Använder du salivstimulerande medel
a. Nej
b. Ja, vilket typ……………………………………….

92. Hur ofta använder du salivstimulerande medel
a. Jag använder inte salivstimulerande
b. Dagligen
c. Varje vecka
d. Någon gång i månaden/aldrig

93. Om du använder salivstimulerande:
Vem har rekommenderat dig att använda detta
a. Jag använder inte salivstimulerande
b. Tandläkare
c. Tandhygienist
d. Sjukvårdspersonal
e. Media
f. Annat………………………………………………
…………………………………………

94. Hur känns ytan på dina tänder
a. Inget speciellt
b. Glatt
c. Sträv
d. Trasiga
e. Annat:…………………………………………

Hur mycket och vad brukar du dricka

	ml	Hur ofta/dag/vecka	Anm
**Cola-drycker:**			
**101.** Light			
**102.** Med socker			
**Annan läsk:**			
**103.** Light			
**104.** Med socker			
**105.** Sportdryck:			
**106.** Äppelcidervinäger:			
**Juice:**			
**107.** Typ			
**Näringsdryck:**			
**108.** Typ			
**Te:**			
**109.** Typ av te:			
**110.** Utan socker			
**111.** Med socker			
**Kaffe:**			
**112.** Utan socker			
**113.** Med socker			
**114.** Mjölk			
**115.** C-vitamin brus			
**116.** Vitamin brus			
**117.** Vatten			
**118. Frukt**	**Ant./dag/vecka**		**Anm.**
**119.** Äpple			
**120.** Päron			
**121.** Apelsin			
**122.** Citron			**Suger ja □ nej □**
**123.** Mandarin			
**124.** Grape			
**125.** Banan			
**126. Annat:**			

127. Har du tidigare, mer än 1 år tillbaka, ätit/druckit mycket av ovanstående
Ange vad och hur mycket………………………………………………………………………………………
………..
……….

	Ja	Nej
**128.** Tror du att vanlig läsk (med socker) kan skada dina tänder		
**129.** Tror du att light läsk (utan vanligt socker) kan skada dina tänder		
**130.** Tror du att fruktjuice kan skada dina tänder		
**131.** Tror du att sportdryck kan skada dina tänder		
**132.** Tror du att frukt kan skada dina tänder		
**133.** Tror du att godis kan skada dina tänder		
**135.** Tycker du att du fått mycket tandbehandling jämfört andra i din ålder		
**136.** Har du blekt dina tänder		
**139.** Behöver du behandling för isningar/smärta i tänderna		
**140.** Har du förtroende för tandvården		
**141.** Tror du att tandborstning efter att du druckit juice kan skada dina tänder		
**142.** Tror du att tandborstning efter att du druckit light läsk kan skada dina tänder		
**144.** Tror du att det kan vara dåligt för munnen att inte äta/dricka		
**145.** Tror du att kräkning kan skada dina tänder		
**146.** Tror du att tandborstning efter kräkning kan skada dina tänder		

Vad dricker du till:

**Vad dricker du till**	**Produkt**	**Hur mycket (ml)**
**147.** Frukost		
**148.** Lunch		
**149.** Middag		
**150.** Kvällen		
**151.** Under natten		
**152.** Mellan måltiderna		
**153.** Under träning		
**154.** Efter träning		
**155.** Hur många mål mat äter du /dag		

**Hur ofta äter du**	Sällan eller Aldrig	En till flera ggr per månad	En till flera ggr per vecka	Dagligen	Flera ggr dagligen
**156.** Godis	1	2	3	4	5
**157.** Kakor/bullar/kex	1	2	3	4	5
**158.** Hård ost	1	2	3	4	5
**159.** Filmjölk/yoghurt	1	2	3	4	5

160. Hur ofta äter du

a. Frukost …………………gånger/vecka
b. Lunch……………………gånger/vecka
c. Middag…………………..gånger/vecka
d. Mellanmål………………gånger/vecka

174. Om du är vegetarian, vilken typ och hur länge har du varit det

a. Jag är inte vegetarian
b. Ja, jag är ……………………
sedan……………………………år/månader
c. Jag har varit vegetarian tidigare
Typ……………………………………..
Tid………………………………år/månader
