# Supplementary material for: Diet and behavioral habits related to oral health in eating disorder patients: a matched case-control study
Source: J Eat Disord. 2020 Feb 27;8:7. doi: 10.1186/s40337-020-0281-z (PMC7045484; doi:10.1186/s40337-020-0281-z)
Supplement: Supplementary file 2 — Additional file 2: Table S1. Drink Intake, Eating and Oral Hygiene Habits in Relatively Good and Bad Disease State Compared to Healthy Controls in vomiting and no vomiting patients1 compared to healthy controls. Table S2. Percentage Distribution of Intake of Dietary Items in Relatively Good and Bad Disease State in vomiting and no vomiting patients1 Compared to Healthy Controls. [file 40337_2020_281_MOESM2_ESM.docx]

**Supplementary Tables for the Vomiting and no Vomiting Groups**

**Table S1** Drink Intake, Eating and Oral Hygiene Habits in Relatively Good and Bad Disease State Compared to Healthy Controls in vomiting and no vomiting patients^1^ compared to healthy controls

|  | Vomiting (n=25) | | |  |  |  |  |  | No vomiting (n=29) | | |  |  |  |  |
| --- | --- | --- | --- | --- | --- | --- | --- | --- | --- | --- | --- | --- | --- | --- | --- |
|  | Relatively good | Bad | Control |  |  |  |  |  | Relatively good | Bad | Control |  |  |  |  |
| **Total Drink Intake (L/yr)** | Mean ±SD | Mean ±SD | Mean ±SD | P | P_1_ | P_2_ | P_3_ |  | Mean ±SD | Mean ±SD | Mean ±SD | P | P_1_ | P_2_ | P_3_ |
| Cola light | 79.9 ±149.2 | 106 ±26 | 6.8 ±19.9 | 0.001 | 0.003 | 0.04 | NS |  | 8.1±23.4 | 26.9 ±103 | 6.4±13.6 | NS | NS | NS | NS |
| Cola regular | 5.9 ±18.0 | 14.2 ±40.2 | 17.2 ±27.8 | 0.006 | NS | NS | NS |  | 11.0 ±27.8 | 10.8 ±55.9 | 27.3 ±39.2 | 0.001 | 0.004 | 0.005 | NS |
| Other carbonated light soft drinks | 28.3 ±109.8 | 42.2 ±129 | 21.2 ±52.1 | NS | NS | NS | NS |  | 0.9 ±3,5 | 0.7 ±3.4 | 3.6 ±11.7 | NS | NS | NS | NS |
| Other carbonated regular soft drinks | 3.1 ±15.6 | 9.4 ±37.5 | 15.3 ±31.9 | 0.002 | 0.04 | NS | NS |  | 7.2 ±33.8 | 6.8 ±33.9 | 25.3 ±68.8 | 0.006 | 0.03 | 0.03 | NS |
| Sport drinks | 0.7 ±3.4 | 0 ±0 | 0 ±0 | NS | NS | NS | NS |  | 0 ±0 | 0 ±0 | 4.5 ±19.5 | NS | NS | NS | NS |
| Apple vinegar | 0.6 ±2.9 | 1.7 ±6.1 | 0 ±0 | NS | NS | NS | NS |  | 0.1 ±0.6 | 0.3 ±1.7 | 1.2 ±6.4 | NS | NS | NS | NS |
| Juice | 24.2 ±43.7 | 34.6 ±59.6 | 50.5 ±50.4 | NS | NS | NS | NS |  | 32.9 ±55.5 | 31.7 ±57.0 | 39.8 ±53.6 | NS | NS | NS | NS |
| Nutrition drinks | 17.5 ±49.2 | 12.4 ±54.9 | 0 ±0 | NS | NS | NS | NS |  | 32.0 ±60.7 | 18.3 ±47.1 | 0 ±0 | 0.002 | 0.01 | NS | NS |
| Tea without sugar | 76.8 ±91.7 | 46.3 ±81.1 | 60.3 ±80.5 | NS | NS | NS | NS |  | 95.2 ±145.8 | 91.9 ±200.8 | 23.1 ±47.2 | NS | 0.03 | NS | NS |
| Tea with sugar | 23.0 ±60.0 | 25.9 ±72.3 | 23.1 ±49.8 | NS | NS | NS | NS |  | 10.8 ±36.7 | 10.2 ±34.5 | 18.0 ±47.7 | NS | NS | NS | NS |
| Coffee without sugar | 138 ±296 | 125 ±297 | 29.8 ±78.4 | NS | NS | NS | NS |  | 60.5 ±115.4 | 56.7 ±133 | 32.9 ±85.3 | NS | NS | NS | NS |
| Coffee with sugar | 7.0 ±32.8 | 6.6 ±32.9 | 7.4 ±36.5 | NS | NS | NS | NS |  | 0.6 ±3.4 | 0.6 ±3.4 | 2.0 ±6.6 | NS | NS | NS | NS |
| Milk | 94.1 ±110 | 69.5 ±122 | 96.5 ±91.5 | NS | NS | NS | NS |  | 85.9 ±80.6 | 69.6 ±82.2 | 116.3 ±121 | 0.005 | NS | 0.04 | 0.02 |
| Water | 376 ±301 | 493 ±532 | 328 ±289 | NS | NS | NS | NS |  | 313 ±314 | 294 ±381 | 294 ±225 | NS | NS | NS | NS |
| Total drink intake (per above) | 880 ±531 | 1005 ±904 | 657 ±322 | NS | NS | NS | NS |  | 652 ±349 | 613 ±455 | 595 ±254 | NS | NS | NS | NS |
| **Soft Drink Consumption** | |  |  |  |  |  |  |  |  |  |  |  |  |  |  |
| Total soft drinks^12^ | 142 ±222 | 205 ±393 | 110 ±93 | NS | NS | NS | NS |  | 59.0 ±112 | 75.8 ±147 | 107 ±102 | 0.02 | 0.01 | 0.04 | NS |
| Total carbonated soft drinks | 117 ±227 | 171 ±381 | 60.4 ±69.4 | NS | NS | NS | NS |  | 27.2 ±67.6 | 45.2 ±118 | 62.6 ±71.4 | 0.006 | 0.02 | NS | NS |
| Total carbonated light soft drinks | 108 ±229 | 147 ±382 | 27.9 ±59.1 | NS | NS | NS | NS |  | 9.0 ±23.4 | 27.6 ±102 | 10.0 ±20.5 | NS | NS | NS | NS |
| Total carbonated regular soft drinks | 9.1 ±32.2 | 23.6 ±75.8 | 32.5 ±40.1 | 0.001 | 0.01 | NS | NS |  | 18.2 ±66.0 | 17.6 ±66.1 | 52.6 ±72.1 | 0.001 | 0.002 | 0.002 | NS |
| Total caffeine-containing drinks^3^ | 330 ±309 | 322 ±425 | 144 ±140 | 0.01 | 0.009 | NS | NS |  | 183 ±174 | 194 ±304 | 110 ±107 | NS | NS | NS | NS |
| **Eating Habits** |  |  |  |  |  |  |  |  |  |  |  |  |  |  |  |
| Number of meals/day | 3.7 ±1.7 | 2.6 ±1.8 | 3.3 ±1.1 | 0.016 | NS | NS | 0.005 |  | 3.8 ±1.4 | 3.3 ±1.8 | 3.5 ±0.9 | 0.03 | NS | NS | 0.002 |
| Number breakfast/week | 5.5 ±2.3 | 3.9 ±3.0 | 6.0 ±1.8 | 0.004 | NS | 0.008 | 0.016 |  | 6.4 ±1.8 | 5.6 ±2.6 | 6.6 ±1.2 | 0.04 | NS | NS | 0.03 |
| Number of lunch/week | 5.3 ±2.3 | 3.7 ±2.4 | 6.6 ±1.1 | 0.001 | 0.03 | 0.001 | 0.008 |  | 6.6 ±1.3 | 5.4 ±2.6 | 6.3 ±1.4 | 0.005 | NS | NS | 0.005 |
| Number of dinner/week | 6.1 ±1.4 | 4.4 ±2.5 | 6.4 ±1.4 | 0.001 | NS | 0.001 | 0.005 |  | 6.8 ±0.9 | 5.6 ±2.6 | 6.5 ±1.7 | NS | NS | NS | 0.03 |
| Number of in-between meals/week | 6.4 ±4.3 | 4.8 ±8.3 | 5.3 ±3.2 | NS | NS | NS | NS |  | 6.2 ±2.8 | 4.9 ±3.4 | 5.0 ±2.9 | NS | NS | NS | NS |
| **No. of fruit intake/week** | |  |  |  |  |  |  |  |  |  |  |  |  |  |  |
| Apples | 6.1 ±6.6 | 6.2 ±15.0 | 4.0 ±6.0 | NS | NS | NS | NS |  | 6.9 ±10.0 | 6.9 ±11.4 | 2.6 ±3.1 | NS | 0.02 | 0.01 | NS |
| Pears | 2.4 ±4.5 | 2.5 ±10.0 | 0.8 ±1.3 | NS | NS | NS | NS |  | 2.2 ±3.9 | 3.2 ±5.2 | 1.0 ±2.0 | NS | NS | NS | NS |
| Citrus fruits | 3.5 ±5.9 | 1.5 ±4.4 | 2.3 ±4.5 | NS | NS | NS | NS |  | 5.2 ±7.3 | 5.9 ±11.1 | 2.0 ±4.3 | NS | NS | NS | NS |
| Bananas | 3.3 ±4.1 | 3.2 ±4.4 | 4.1 ±5.4 | NS | NS | NS | NS |  | 5.4 ±12.3 | 3.0 ±6.6 | 3.2 ±3.4 | NS | NS | NS | 0.03 |
| **Oral Hygiene Habits** | |  |  |  |  |  |  |  |  |  |  |  |  |  |  |
| No. of brushing times/day | 2.3 ±0.8 | 2.2 ±1.2 | 2.1 ±0.6 | NS | NS | NS | NS |  | 2.4 ±0.7 | 2.9 ±1.8 | 2.0 ±0.4 | 0.004 | 0.015 | 0.03 | 0.02 |
| Toothbrushing min/time | 2.7 ±1.8 | 2.6 ±1.8 | 3.0 ±1.6 | NS | NS | NS | NS |  | 3.8 ±4.1 | 4.2 ±4.7 | 2.8 ±1.5 | NS | NS | NS | NS |
| Cm tooth paste /brushing | 1.5 ±0.6 | 1.5 ±0.6 | 1.4 ±0.6 | NS | NS | NS | NS |  | 1.5 ±1.1 | 1.5 ±1.1 | 1.8 ±1.2 | NS | NS | NS | NS |

P refers to differences between the three groups by Friedman test. P_1_ refers to difference between relatively good vs. control; P_2_ bad vs. control; P_3_ relatively good vs. bad (Wilcoxon Signed Rank Test)

^1^ Based on the question “Are you presently or previously been engaged in self-induced vomiting”

^2^ Carbonated beverages (light and regular), sport drinks, juice

^3^ Cola-type drinks, tea, coffee

**Table S2** Percentage Distribution of Intake of Dietary Items in Relatively Good and Bad Disease State in vomiting and no vomiting patients^1^ Compared to Healthy Controls.

|  | Vomiting (n=25) | | | | | |  | | | |  | No vomiting(n=29) | | | | | |  |  |  |  |
| --- | --- | --- | --- | --- | --- | --- | --- | --- | --- | --- | --- | --- | --- | --- | --- | --- | --- | --- | --- | --- | --- |
|  | Relatively good | | Bad | | Control | |  | | | |  | Relatively good | | Bad | | Control | |  |  |  |  |
|  | 1 | 2 | 1 | 2 | 1 | 2 | P | P_1_ | P_2_ | P_3_ |  | 1 | 2 | 1 | 2 | 1 | 2 | P | P_1_ | P_2_ | P_3_ |
| Sweets | 36 | 64 | 48 | 52 | 24 | 76 | NS | NS | NS | NS |  | 38 | 62 | 55 | 45 | 10 | 90 | 0.001 | 0.02 | 0.002 | NS |
| Sweet biscuits, buns | 44 | 56 | 56 | 11 | 24 | 76 | NS | NS | NS | NS |  | 48 | 52 | 76 | 24 | 14 | 86 | 0.001 | 0.02 | 0.001 | 0.00 |
| Hard cheese | 48 | 52 | 56 | 44 | 20 | 80 | 0.08 | NS | 0.01 | NS |  | 55 | 45 | 61 | 39 | 43 | 57 | NS | NS | NS | NS |
| Yoghurt/sour milk | 20 | 80 | 36 | 64 | 28 | 72 | NS | NS | NS | NS |  | 21 | 79 | 35 | 65 | 14 | 86 | NS | NS | NS | NS |

1= Never or seldom; 2= More than one intake/month. P refers to differences between the three groups by Friedman test. P_1_ refers to difference between self-perceived relatively good vs. control; P_2_ bad vs. control; P_3_ relatively good vs. bad (McNemar's Test)
